# Association between decreases in serum uric acid levels and unfavorable outcomes after ischemic stroke: A multicenter hospital-based observational study

**DOI:** 10.1371/journal.pone.0287721

**Published:** 2023-06-29

**Authors:** Kuniyuki Nakamura, Kana Ueki, Ryu Matsuo, Takuya Kiyohara, Fumi Irie, Yoshinobu Wakisaka, Tetsuro Ago, Masahiro Kamouchi, Takanari Kitazono, on behalf of the Fukuoka Stroke Registry Investigators

**Affiliations:** 1 Department of Medicine and Clinical Science, Graduate School of Medical Sciences, Kyushu University, Fukuoka, Japan; 2 Department of Health Care Administration and Management, Graduate School of Medical Sciences, Kyushu University, Fukuoka, Japan; 3 Center for Cohort Studies, Graduate School of Medical Sciences, Kyushu University, Fukuoka, Japan; Foshan Sanshui District People’s Hospital, CHINA

## Abstract

**Background:**

The association between clinical outcomes in ischemic stroke patients and decreases in serum uric acid levels, which often occur during the acute phase, remains unknown. Herein, we aimed to investigate the association using a large-scale, multicenter stroke registry.

**Methods:**

We analyzed 4,621 acute ischemic stroke patients enrolled in the Fukuoka Stroke Registry between June 2007 and September 2019 whose uric acid levels were measured at least twice during hospitalization (including on admission). The study outcomes were poor functional outcome (modified Rankin Scale score ≥3) and functional dependence (modified Rankin Scale score 3–5) at 3 months after stroke onset. Changes in uric acid levels after admission were evaluated using a decrease rate that was classified into 4 sex-specific grades ranging from G1 (no change/increase after admission) to G4 (most decreased). Multivariable logistic regression analyses were used to assess the associations between decreases in uric acid levels and the outcomes.

**Results:**

The frequencies of the poor functional outcome and functional dependence were lowest in G1 and highest in G4. The odds ratios (95% confidence intervals) of G4 were significantly higher for poor functional outcome (2.66 [2.05–3.44]) and functional dependence (2.61 [2.00–3.42]) when compared with G1 after adjusting for confounding factors. We observed no heterogeneity in results for subgroups categorized according to age, sex, stroke subtype, neurological severity, chronic kidney disease, or uric acid level on admission.

**Conclusions:**

Decreases in serum uric acid levels were independently associated with unfavorable outcomes after acute ischemic stroke.

## Introduction

Stroke is a leading cause of death and disability worldwide, and methods that can consistently and effectively prevent poor functional outcomes after acute ischemic stroke remain elusive even in the era of thrombolysis and endovascular thrombectomy. Previous studies have already identified various predictors of poor functional outcomes after stroke, including hypertension [1], poor glycemic control [2], insulin resistance [3], smoking habit [4], and chronic kidney disease (CKD) [5]. However, many patients still suffer from poor functional outcomes following stroke despite the control of these risk factors. Accordingly, there is an urgent need to clarify what other factors may contribute to unfavorable outcomes as residual risks.

Hyperuricemia is an independent predictor of cardiovascular events. A previous meta-analysis demonstrated that hyperuricemia was significantly associated with a greater risk of both stroke incidence and mortality [6]. Hyperuricemia is not only a comorbid disease that accompanies numerous cardiovascular risk factors (e.g., hypertension, diabetes mellitus, obesity, CKD, and smoking/alcohol habits), but there is a growing concern that uric acid (UA) itself plays a pro-inflammatory role. High serum UA levels can lead to the crystallization of monosodium urate, thereby inducing inflammation mediated by interleukin-1 and the NLR family pyrin domain–containing 3 inflammasome, which ultimately results in atherosclerosis [7, 8].

On the other hand, other studies have noted the beneficial roles of UA in the central nervous system [9, 10]. UA, a final enzymatic product of purine metabolism catalyzed by xanthine oxidase, has an antioxidant capacity via the scavenging of oxidative stress agents [11]. Because ischemia/reperfusion injury–induced reactive oxygen species contribute to neuronal cell death, the antioxidant effect of UA may favor neuronal survival. However, the association between UA levels and clinical outcomes in ischemic stroke patients remains controversial, with a variety of studies showing positive [12–17], negative [18–20], or U-shaped [21–23] relationships. These discrepant findings may be due to the changes in UA levels during the acute phase of ischemic stroke. Few studies have examined whether dynamic changes in UA levels affect clinical outcomes after stroke onset, with only one study reporting that a decrease in UA levels in the first week after stroke correlates with larger infarct volumes and increased stroke severity [24]. In other words, it remains unknown as to whether decreases in UA levels during the acute phase specifically affect functional and neurological outcomes in patients with ischemic stroke.

With a focus on dynamic changes in UA levels during the acute phase of ischemic stroke, this study aimed to investigate the association between decreases in serum UA levels during hospitalization and clinical outcomes using a large-scale, multicenter registry of acute ischemic stroke patients in Japan.

## Materials and methods

### Data availability

Anonymized data are available from the corresponding author upon reasonable request.

### Standard protocol approvals, registrations, and patient consent

The Fukuoka Stroke Registry is a prospective multicenter hospital-based registry (UMIN-CTR 000000800) [2] that enrolls stroke patients who are hospitalized within a week of onset in 7 participating hospitals located in Fukuoka Prefecture, Japan. The participating hospitals were Kyushu University Hospital (Fukuoka, Japan), National Hospital Organization Kyushu Medical Center (Fukuoka, Japan), National Hospital Organization Fukuoka–Higashi Medical Center (Koga, Japan), Fukuoka Red Cross Hospital (Fukuoka, Japan), St. Mary’s Hospital (Kurume, Japan), Steel Memorial Yawata Hospital (Kitakyushu, Japan), and Japan Labour Health and Welfare Organization Kyushu Rosai Hospital (Kitakyushu, Japan). The institutional review boards of all participating hospitals approved the study protocol. Written informed consent was obtained from all patients or their family members. Stroke was defined as the sudden onset of a nonconvulsive and focal neurologic deficit. All patients underwent brain computed tomography, magnetic resonance imaging, or both within 24 hours of hospitalization. Ischemic stroke included transient ischemic attack and diffusion-weighted image-negative cases.

### Participants

In total, 15,569 patients with acute ischemic stroke were registered in the Fukuoka Stroke Registry from June 2007 to September 2019. We excluded 3,386 patients who showed disabilities in their daily activities before stroke onset (modified Rankin Scale [mRS] score ≥2) and 250 patients who were lost to follow-up by 3 months after onset. To evaluate changes in UA levels, we focused on patients who had undergone blood tests on admission and at ≥1 time points during hospitalization. We excluded 7,309 patients with missing UA level data and 3 patients whose UA levels were considered outliers (>1784 μmol/L [30 mg/dL]) based on the distribution of all measured UA levels in all enrolled patients. After applying these exclusion criteria, we obtained a final study population of 4,621 patients with acute ischemic stroke for analysis (S1 Fig).

### Clinical assessments

We assessed the patients’ characteristics, including age, sex, body mass index (BMI), estimated glomerular filtration rate (eGFR), cardiovascular risk factors (hypertension, diabetes mellitus, dyslipidemia, atrial fibrillation, smoking habit, and alcohol habit), coronary artery disease, CKD (eGFR <60 ml/min/1.73 m^2^ and/or proteinuria), previous history of stroke, length of hospital stay, and administration of antihyperuricemics during hospitalization; these characteristics were selected as potential influencing factors on clinical outcomes identified in previous studies [1–5]. Using the Trial of ORG 10172 in Acute Stroke Treatment criteria [25], ischemic stroke was classified into the following 4 subtypes: cardioembolism, large artery atherosclerosis, small vessel occlusion, and other causes. Trained stroke neurologists assessed each patient’s National Institutes of Health Stroke Scale (NIHSS) score on admission and during hospitalization as an indicator of neurological severity. Acute reperfusion therapy included intravenous thrombolysis with recombinant tissue-type plasminogen activator and endovascular therapy.

### Study outcomes

The primary study outcomes were poor functional outcome and functional dependence at 3 months after stroke onset. Poor functional outcome was defined as an mRS score of 3 to 6, whereas functional dependence was defined as an mRS score of 3 to 5 (excluding death) [3]. The secondary study outcomes were neurological improvement and neurological deterioration during hospitalization, all-cause death within 3 months of onset, and stroke recurrence within 3 months of onset. Neurological improvement was defined as a ≥4-point decrease in NIHSS score during hospitalization (vs. score on admission) or a score of zero at discharge [2, 3]. Neurological deterioration was defined as a ≥1-point increase in NIHSS score during hospitalization (vs. score on admission) [2]. The mRS score at 3 months, death within 3 months, and stroke recurrence within 3 months were evaluated by trained and certified research nurses via telephone using a standardized structured questionnaire.

### Measurements of serum uric acid levels

Blood samples were collected at day 0 (on admission) of hospitalization and at ≥1 subsequent arbitrary time points during days 1–3, days 4–6, days 7–10, or day 11 or later. Serum UA concentrations were measured using an enzymatic method.

Changes in UA levels during hospitalization (relative to levels on admission) were evaluated using the UA decrease rate, which was calculated using the following formula: [(UA at day 0—minimum UA during hospitalization)/UA at day 0] × 100. These UA decrease rates were categorized into 4 grades—designated G1 to G4—for women (G1, 0%; G2, 0.01–11.11%; G3, 11.12–23.53%; and G4, >23.53%) and men (G1, 0%; G2, 0.01–9.21%; G3, 9.22–19.79%; and G4, >19.79%).

In addition, the UA levels on admission were categorized into quintiles [26] for women (Q1, ≤228 μmol/L [3.8 mg/dL]; Q2, 229–270 μmol/L [3.9–4.5 mg/dL]; Q3, 271–312 μmol/L [4.6–5.2 mg/dL]; Q4, 313–365 μmol/L [5.3–6.1 mg/dL]; and Q5, >365 μmol/L [6.1 mg/dL]) and men (Q1, ≤276 μmol/L [4.6 mg/dL]; Q2, 277–324 μmol/L [4.7–5.4 mg/dL]; Q3, 325–371 μmol/L [5.5–6.2 mg/dL]; Q4, 372–431 μmol/L [6.3–7.2 mg/dL]; and Q5, >431 μmol/L [7.2 mg/dL]).

### Statistical analysis

Paired *t*-tests were used to assess the temporal changes in UA levels during hospitalization. Trends in baseline characteristics according to UA decrease rate grade were evaluated using the Jonckheere–Terpstra trend test or the Cochran–Armitage trend test, as appropriate.

Logistic regression models were constructed to calculate the odds ratios (ORs) and 95% confidence intervals (CIs) for the UA decrease rate grades (G1 to G4) and for quintiles of UA levels on admission (Q1 to Q5) after adjusting for potential confounding factors. First, we used models that adjusted for age and sex only. Next, we used multivariable models that adjusted for a variety of quantitative variables (age, BMI, eGFR, mRS score before stroke onset, NIHSS score on admission, length of hospital stay, and serum UA level on admission) and categorical variables (sex, hypertension, diabetes mellitus, dyslipidemia, atrial fibrillation, smoking habit, alcohol habit, stroke subtype, and acute reperfusion therapy). These variables were selected due to their association with stroke functional outcomes and potential impact on UA levels as reported in previous studies [1, 3, 4, 27, 28]. To evaluate potential heterogeneity, we conducted subgroup analyses in which patients were divided into subgroups according to age (<75 or ≥75 years), sex (women or men), stroke subtype (cardioembolic or non-cardioembolic), neurological severity (NIHSS score on admission <5 or ≥5), CKD (absence or presence), and UA level on admission (≤292 μmol/L [4.9 mg/dl] or >292 μmol/L for women; ≤351 μmol/L [5.9 mg/dL] or >351 μmol/L for men). *P* values for heterogeneity were calculated by adding the interaction term of the UA decrease rate grade × subgroup to the multivariable models.

We performed a sensitivity analysis on the associations between decreases in serum UA levels and modified primary study outcomes at 3 months; for this analysis, poor functional outcome was defined as mRS 2–6 (instead of mRS 3–6) and functional dependence was defined as mRS 2–5 (instead of mRS 3–5). We also evaluated these associations when participants were categorized according to the difference in serum UA levels between admission and nearest to discharge. Moreover, sensitivity analyses were performed for patients who were admitted within 24 hours of stroke onset and patients who were not administered antihyperuricemics during hospitalization. We also conducted a sensitivity analysis in which serum albumin levels (indicating nutritional status) and hematocrit levels (indicating blood fluid volume) on admission were added to the multivariable models as covariates.

Statistical analyses were performed using JMP 16 (SAS Institute Inc, Cary, NC, USA) and STATA 15 (StataCorp LP, College Station, TX, USA) software. A two-tailed value of *P* < 0.05 was considered statistically significant.

## Results

### Temporal profile of serum uric acid levels

**Fig 1:**
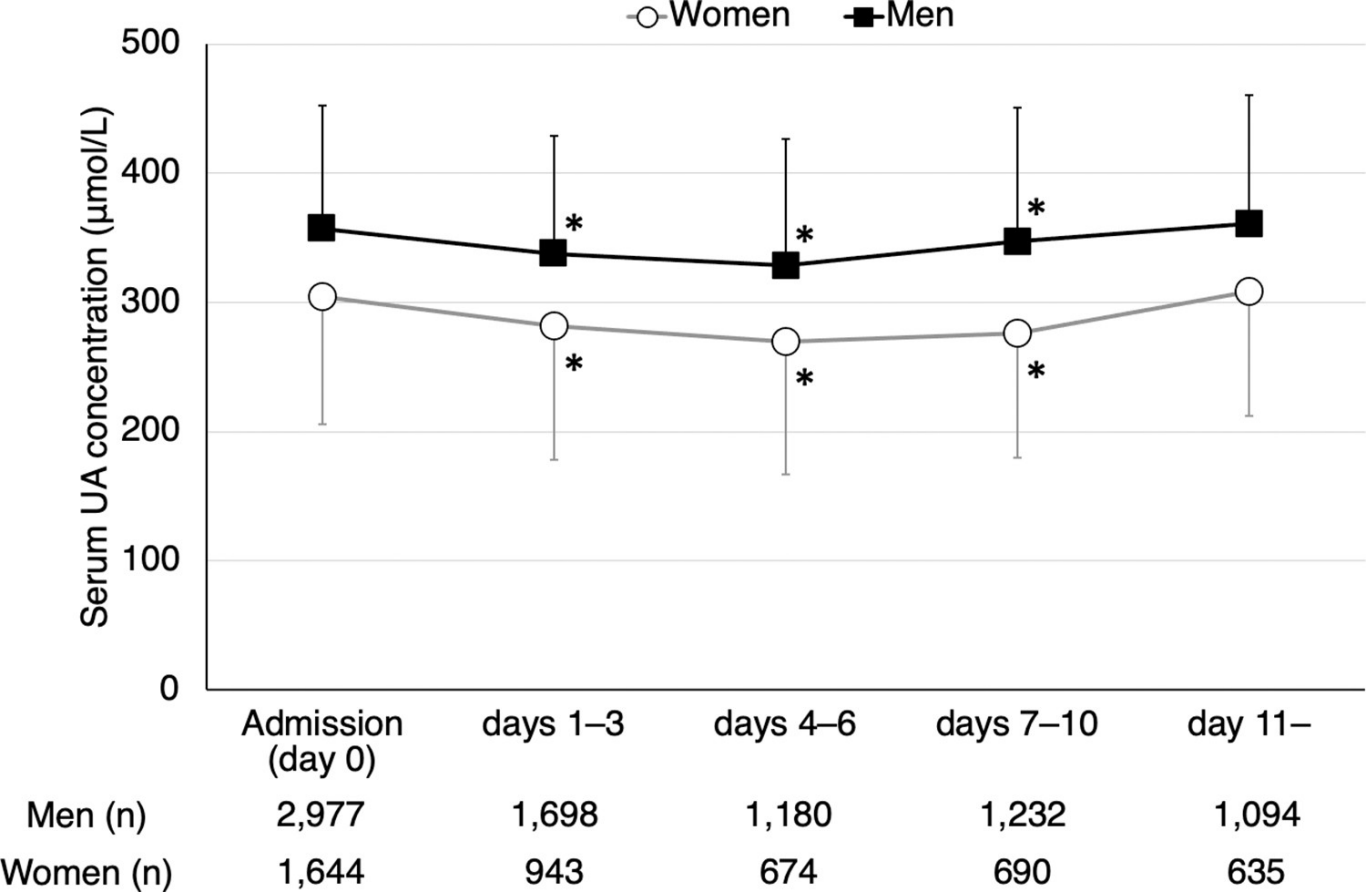
Temporal profile of serum UA levels during hospitalization. The serum UA levels on admission (day 0), days 1–3, days 4–6, days 7–10, and day 11 or later are presented according to sex. The data are expressed according to sex as mean values ± standard deviation. * *P* < 0.05 vs. serum UA levels on admission by paired *t*-test. UA indicates uric acid.

Firstly, we examined the temporal profile of UA levels during hospitalization in the 4,621 ischemic stroke patients according to sex (Fig 1). The overall UA level on admission (mean ± standard deviation) was lower in women (304.1 ± 98.2 μmol/L) than in men (357.2 ± 95.6 μmol/L). The UA levels at days 1–3, days 4–6, and days 7–10 were significantly lower than those on admission in either sex. No significant differences were observed in the UA levels between on admission and at day 11 or later.

### Patient characteristics

The mean age of our study patients was 70.1 ± 12.2 years, and 64.4% were men. Table 1 summarizes the patient characteristics according to UA decrease rate grade. Higher UA decrease rates (i.e., from G1 to G4) were positively associated with patient age, UA level on admission, NIHSS score on admission, length of hospital stay, and the frequencies of atrial fibrillation, alcohol habit, CKD, cardioembolism, and reperfusion therapy. In contrast, higher UA decrease rates were negatively associated with the proportion of men, BMI, eGFR, and the frequencies of diabetes mellitus, dyslipidemia, smoking habit, and coronary artery disease.

**Table 1:** Patient characteristics according to serum UA decrease rate grade.

	G1, n = 1556	G2, n = 1023	G3, n = 1024	G4, n = 1018	*P*_trend_
UA level decrease (%), women	0	0.01–11.11	11.12–23.53	> 23.53	
UA level decrease (%), men	0	0.01–9.21	9.22–19.79	> 19.79	
Age (years), mean ± SD	68.4±12.3	70.1±12.0	70.0±12.1	72.6±12.0	**<0.001**
Men, n (%)	1053 (67.7)	642 (62.8)	646 (63.1)	636 (62.5)	**0.006**
UA level on admission (μmol/L), mean ± SD					
Women	268.8±83.4	309.7±79.6	313.3±96.4	335.8±119	**<0.001**
Men	324.1±84.4	361.0±84.5	375.5±89.9	389.7±111.1	**<0.001**
BMI (kg/m^2^), mean ± SD	23.6±3.7	23.6±3.7	23.4±3.6	22.6±3.8	**<0.001**
eGFR (mL/min/1.73 m^2^), mean ± SD	66.4±28.2	65.7±23.0	67.1±23.6	64.3±22.7	**0.001**
Risk factors, n (%)					
Hypertension	1265 (81.3)	861 (84.2)	849 (82.9)	822 (80.7)	0.79
Diabetes mellitus	543 (34.9)	344 (33.6)	334 (32.6)	305 (30.0)	**0.01**
Dyslipidemia	945 (60.7)	612 (59.8)	635 (62.0)	538 (52.8)	**0.002**
Atrial fibrillation	269 (17.3)	203 (19.8)	205 (20.0)	311 (30.6)	**<0.001**
Smoking habit	973 (62.5)	601 (58.7)	581 (56.7)	561 (55.1)	**<0.001**
Alcohol habit	560 (36.0)	375 (36.7)	434 (42.4)	400 (39.3)	**0.01**
Coronary artery disease, n (%)	249 (16.0)	146 (14.3)	135 (13.2)	137 (13.5)	**0.04**
Chronic kidney disease, n (%)	633 (40.7)	440 (43.0)	457 (44.6)	499 (49.0)	**<0.001**
Previous history of stroke, n (%)	250 (16.1)	158 (15.4)	160 (15.6)	158 (15.5)	0.72
Stroke subtypes, n (%)					
Cardioembolism	226 (14.5)	179 (17.5)	189 (18.5)	289 (28.4)	**<0.001**
Large artery atherosclerosis	229 (14.7)	166 (16.2)	198 (19.3)	195 (19.2)	
Small vessel occlusion	498 (32.0)	328 (32.1)	332 (32.4)	233 (22.9)	
Other causes	603 (38.8)	350 (34.2)	305 (29.8)	301 (29.6)	
Reperfusion therapy, n (%)	109 (7.0)	97 (9.5)	147 (14.4)	221 (21.7)	**<0.001**
NIHSS score on admission, median (IQR)	2 (1–4)	2 (1–4)	3 (1–5)	5 (2–12)	**<0.001**
Length of hospital stay (days), median (IQR)	16 (11–22)	16 (12–22)	19 (14–25.75)	24 (17–34)	**<0.001**
Antihyperuricemic use during hospitalization	181 (11.6)	93 (9.1)	105 (10.3)	143 (14.0)	0.11

The decrease rates of serum UA levels were calculated between those at day 0 and the minimum value during hospitalization, and were categorized into 4 sex-specific grades (G1 to G4).

BMI indicates body mass index; eGFR, estimated glomerular filtration rate; IQR, interquartile range; NIHSS, National Institutes of Health Stroke Scale; *P*_trend_, *P* for trend; SD, standard deviation; and UA, uric acid.

### Association between decreases in serum uric acid levels and clinical outcomes

The frequencies of poor functional outcome and functional dependence at 3 months after stroke onset were lowest in G1 (no change or increase in UA levels after admission) and highest in G4 (Table 2). Therefore, G1 was used as the reference category in the subsequent analyses. The age- and sex-adjusted ORs and multivariable-adjusted ORs of G3 and G4 were significantly higher (ref: G1) for both poor functional outcome and functional dependence at 3 months (Table 2). These associations were preserved even when poor functional outcome and functional dependence were defined as mRS scores of 2–6 and 2–5, respectively (S1 Table). Similarly, when participants were categorized according to the difference in serum UA levels between admission and nearest to discharge, the group with decreased UA still showed poorer outcomes (S2 Table). However, decreases in UA levels were not significantly associated with all-cause death or stroke recurrence within 3 months (S3 Table). To explore whether decreases in UA levels affect neurological outcomes during the acute phase of ischemic stroke, we evaluated the changes in NIHSS scores during hospitalization (Table 3). The age- and sex-adjusted ORs and multivariable-adjusted ORs for neurological improvement were significantly lower in G4, whereas the ORs for neurological deterioration were significantly higher in G3 and G4 (ref: G1).

**Table 2:** Associations between decreases in serum UA levels and functional outcomes at 3 months.

		Age- and sex-adjusted			Multivariable-adjusted		
	Events/total (%)	OR (95% CI)	*P*	*P*_trend_	OR (95% CI)	*P*	*P*_trend_
**Poor functional outcome at 3 months**							
G1	201/1556 (12.9)	1.00 (reference)		**<0.001**	1.00 (reference)		**<0.001**
G2	143/1023 (14.0)	0.98 (0.78–1.25)	0.90		1.06 (0.80–1.40)	0.70	
G3	221/1024 (21.6)	1.75 (1.41–2.17)	**<0.001**		1.51 (1.16–1.96)	**0.002**	
G4	474/1018 (46.6)	5.31 (4.34–6.49)	**<0.001**		2.66 (2.05–3.44)	**<0.001**	
**Functional dependence at 3 months**							
G1	178/1533 (11.6)	1.00 (reference)		**<0.001**	1.00 (reference)		**<0.001**
G2	135/1015 (13.3)	1.05 (0.82–1.34)	0.69		1.10 (0.83–1.47)	0.51	
G3	201/1004 (20.0)	1.81 (1.44–2.26)	**<0.001**		1.48 (1.13–1.95)	**0.005**	
G4	429/973 (44.1)	5.40 (4.38–6.65)	**<0.001**		2.61 (2.00–3.42)	**<0.001**	

Poor functional outcome and functional dependence were defined as mRS scores of 3–6 and 3–5, respectively, at 3 months after stroke onset. G1 to G4 indicate the grades of serum UA decrease rates. The multivariable models adjusted for patient age, sex, modified Rankin Scale score before stroke onset, body mass index, acute reperfusion therapy, National Institutes of Health Stroke Scale score on admission, stroke subtype, hypertension, diabetes mellitus, dyslipidemia, atrial fibrillation, smoking habit, alcohol habit, estimated glomerular filtration rate, length of hospital stay, and serum UA level on admission.

CI indicates confidence interval; OR, odds ratio; *P*_trend_, *P* for trend; and UA, uric acid.

**Table 3:** Associations between decreases in serum UA levels and neurological outcomes during hospitalization.

		Age- and sex-adjusted			Multivariable-adjusted		
	Events/total (%)	OR (95% CI)	*P*	*P*_trend_	OR (95% CI)	*P*	*P*_trend_
**Neurological improvement**							
G1	887/1556 (57.0)	1.00 (reference)		**<0.001**	1.00 (reference)		**<0.001**
G2	569/1023 (55.6)	0.95 (0.81–1.11)	0.52		0.93 (0.79–1.10)	0.42	
G3	543/1024 (53.0)	0.85 (0.73–1.00)	0.052		0.87 (0.73–1.03)	0.10	
G4	474/1018 (46.6)	0.67 (0.57–0.79)	**<0.001**		0.66 (0.54–0.79)	**<0.001**	
**Neurological deterioration**							
G1	64/1556 (4.1)	1.00 (reference)		**<0.001**	1.00 (reference)		**<0.001**
G2	51/1023 (5.0)	1.17 (0.80–1.70)	0.43		1.21 (0.82–1.79)	0.33	
G3	80/1024 (7.8)	1.90 (1.35–2.66)	**<0.001**		1.68 (1.18–2.41)	**0.004**	
G4	160/1018 (15.7)	3.89 (2.87–5.28)	**<0.001**		2.85 (2.02–4.02)	**<0.001**	

Neurological improvement and deterioration were defined as a ≥4-point decrease in NIHSS score during hospitalization or a score of zero at discharge and a ≥1-point increase in NIHSS score during hospitalization, respectively. G1 to G4 indicate the grades of serum UA decrease rates. The multivariable models adjusted for patient age, sex, modified Rankin Scale score before stroke onset, body mass index, acute reperfusion therapy, NIHSS score on admission, stroke subtype, hypertension, diabetes mellitus, dyslipidemia, atrial fibrillation, smoking habit, alcohol habit, estimated glomerular filtration rate, length of hospital stay, and serum UA level on admission.

CI indicates confidence interval; NIHSS, National Institutes of Health Stroke Scale; OR, odds ratio; *P*_trend_, *P* for trend; and UA, uric acid.

### Association between serum uric acid levels on admission and clinical outcomes

The patient characteristics according to the quintiles of UA levels on admission are shown in S4 Table. Higher UA levels on admission (i.e., from Q1 to Q5) were negatively associated with eGFR and the frequency of diabetes mellitus, but were positively associated with BMI, use of antihyperuricemics during hospitalization, and the frequencies of hypertension, dyslipidemia, atrial fibrillation, CKD, alcohol habit, and cardioembolism. The multivariable-adjusted logistic regression analyses found that UA levels on admission were not significantly associated with poor functional outcome or functional dependence at 3 months (S5 Table).

### Subgroup analysis

**Fig 2:**
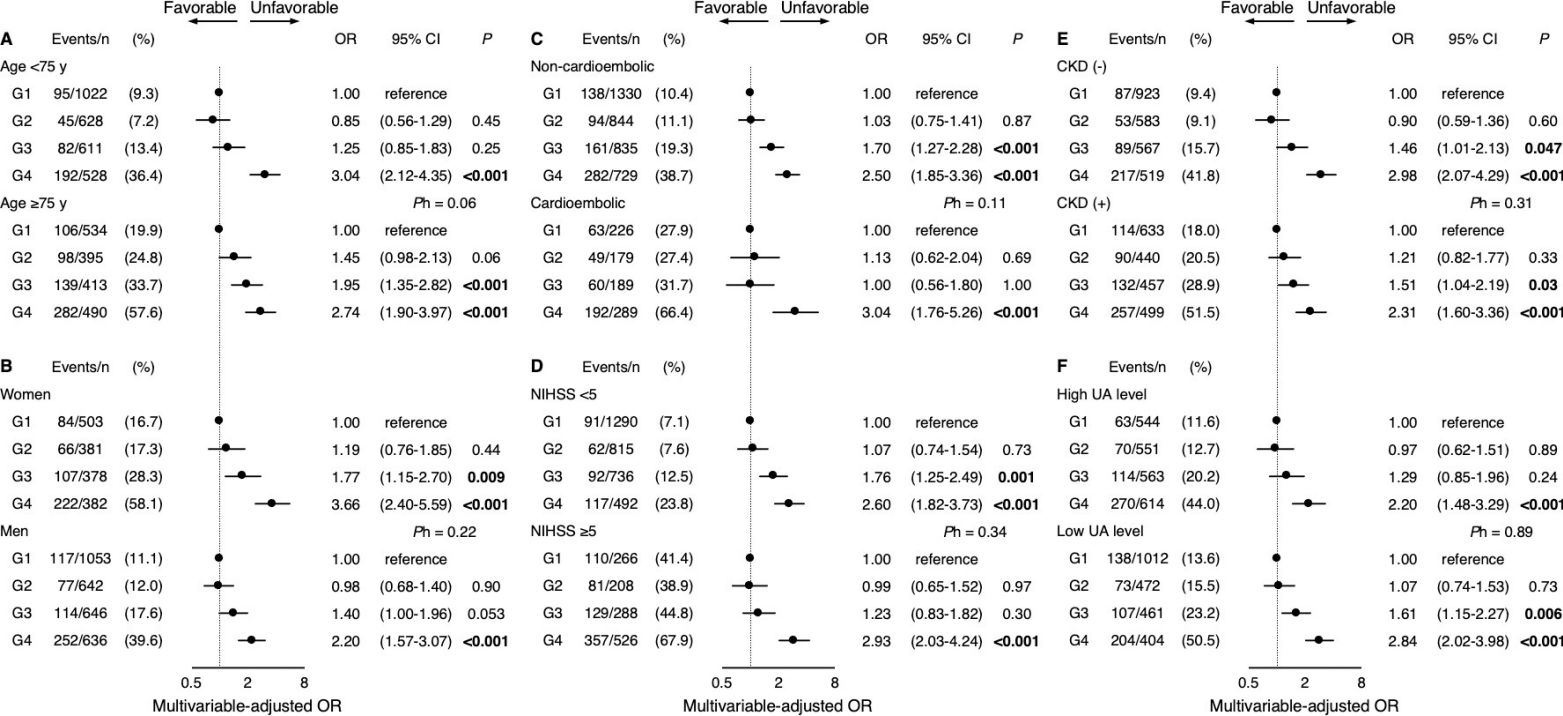
Subgroup analyses of the association between decreases in serum UA levels and poor functional outcome at 3 months. The ORs and 95% CIs of poor functional outcome (defined as an mRS score of 3–6 at 3 months) are shown according to UA decrease rate grade (G1 to G4) in each subgroup. The subgroups included (**A**) age (<75 or ≥75 years), (**B**) sex (women or men), (**C**) stroke subtype (non-cardioembolic or cardioembolic), (**D**) neurological severity (NIHSS <5 or ≥5), (**E**) CKD (presence or absence), and (**F**) UA level on admission (<292 or ≥292 μmol/L for women, <351 or ≥351 μmol/L for men). The multivariable models adjusted for patient age, sex, modified Rankin Scale score before stroke onset, body mass index, acute reperfusion therapy, NIHSS score on admission, stroke subtype, hypertension, diabetes mellitus, dyslipidemia, atrial fibrillation, smoking habit, alcohol habit, estimated glomerular filtration rate, length of hospital stay, and serum UA level on admission. *P* values for heterogeneity (*P*h) were calculated by adding the interaction term of UA decrease rate grade × subgroup to the multivariable models. CI indicates confidence interval; CKD, chronic kidney disease; NIHSS, National Institutes of Health Stroke Scale; OR, odds ratio; and UA, uric acid.

We performed subgroup analyses to determine whether the associations between decreases in UA levels and clinical outcomes differed according to the following factors: age, sex, stroke subtype, neurological severity, CKD, and UA level on admission. In general, we found no heterogeneities between decreases in UA levels and these factors for poor functional outcome, functional dependence, neurological improvement, or neurological deterioration (Fig 2, S2–S4 Figs). The only significant difference was found between sexes in neurological deterioration.

### Sensitivity analysis

As shown in Fig 1, UA levels fluctuate during the acute phase of stroke. Therefore, we assessed the association between decreases in UA levels and poor functional outcome in patients admitted within 24 hours of stroke onset (S6 Table). The age- and sex-adjusted ORs and multivariable-adjusted ORs of G3 and G4 were significantly higher (ref: G1) for both poor functional outcome and functional dependence at 3 months.

Next, we conducted a sensitivity analysis to exclude the potential effects of antihyperuricemic use (S7 Table). By focusing on patients who were not administered these agents during hospitalization, we found that the age- and sex-adjusted ORs and multivariable-adjusted ORs of G3 and G4 were significantly higher (ref: G1) for both poor functional outcome and functional dependence at 3 months.

Finally, we included serum albumin and hematocrit levels on admission into the multivariable model as indicators of nutritional status and body fluid volume, respectively (S8 Table). The multivariable-adjusted ORs of G3 and G4 were significantly higher (ref: G1) for both poor functional outcome and functional dependence at 3 months.

## Discussion

In this multicenter stroke registry study, we found that ischemic stroke patients who experienced decreases in serum UA levels during the acute phase had unfavorable functional outcomes at 3 months even after adjusting for confounding factors such as comorbidities, BMI, kidney function, stroke subtype, neurological severity, and UA level on admission. Furthermore, decreases in UA levels were also associated with poor neurological improvement and neurological deterioration during hospitalization, but not with all-cause death or stroke recurrence without 3 months. Our results indicate that UA may confer a neuroprotective effect during the acute phase of ischemic stroke.

Previous studies have not reached a coherent conclusion regarding the association between UA levels and functional outcomes after ischemic stroke. One reason for this discrepancy may be the lack of standardization in study design, outcomes, and scale. For example, numerous previous studies did not consider neurological severity at stroke onset [14–16, 21, 23], BMI [14–18, 20, 21, 23, 24], or kidney function [14–18, 24]. Moreover, many of these studies did not consider sex differences in UA distribution. Thus, we adjusted for these confounding factors in our study, which demonstrated that there were no significant associations between UA level on admission and clinical outcomes in acute ischemic stroke patients. On the other hand, decreases in UA levels in the acute phase after stroke onset were associated with poor clinical outcomes. These observations suggest that a “lowering UA level” may have a greater impact on unfavorable outcomes after ischemic stroke than a “low UA level”.

Our finding that decreases in UA levels were associated with poor neurological improvement and neurological deterioration may indicate a neuroprotective role of UA in acute ischemic stroke. UA administration has been reported to protect neurons against oxyradical-mediated damage in a rat ischemic stroke model [27] and to improve stroke outcomes in patients with intravenous thrombolytic therapy [28]. It has also been demonstrated that UA has an antioxidant effect that may inhibit the development and progression of other central nervous system diseases, such as multiple sclerosis, Alzheimer’s disease, and Parkinson’s disease [9, 10]. Therefore, decreases in UA levels may be associated with the removal of these neuroprotective effects, resulting in neurological deterioration after ischemic stroke. In contrast, a recent study of hypouricemic patients suggested that excessively low UA levels are associated with endothelial dysfunction due to oxidative stress [29]. Therefore, endothelial dysfunction may be another contributing factor for poor outcomes in stroke patients with low UA levels.

On the other hand, severe brain injury could induce a decrease in UA levels after ischemic stroke. A previous report suggested that inflammation in multiple sclerosis patients can induce hypouricemia through the overconsumption of UA as a scavenger of excessive oxidative stress [30]. UA levels have also been reported to decline in patients with traumatic brain injury or after cerebral tumor surgery [31]. However, there has been no clear evidence that elucidates the mechanism underlying the effects of ischemic injury on UA levels, such as from studies that clarify the activities of xanthine oxidase and urate transporters using an animal stroke model.

UA levels during the acute phase of stroke are likely to change due to body fluid volume or other factors [24], such as the administration of infusion therapy and/or a decrease in food intake during hospitalization. Low UA levels may be associated with malnutrition [32], which is a frequent condition in patients with dementia, and the resultant frailty could induce neurological deterioration. Indeed, a comprehensive liquid chromatography–mass spectrometry metabolomic analysis demonstrated that UA levels were decreased in patients with frailty [33]. Nevertheless, our study showed that the associations between UA levels and functional outcomes were maintained after adjusting for serum albumin and hematocrit levels as indicators of nutritional status and body fluid volume, respectively, suggesting that the influence of these factors is relatively small.

Interestingly, although the UA level distribution differed between men and women, decreases in UA levels were similarly associated with poor functional outcome in both sexes. In contrast, the effect on neurological deterioration was greater in women than in men. This suggests that the optimal value of UA levels for neuroprotection may differ by sex. Hyperuricemia is typically defined as a UA level greater than 405 μmol/L (6.8 mg/dL) in both sexes based on the saturation point of monosodium urate [34]. This definition was also supported by a meta-analysis showing similar effects of UA increments on the development of ischemic stroke in men and women [35]. Nevertheless, there remains uncertainty as to the validity and basis of the definition of hypouricemia in men and women. Taking into consideration our observed differences in neuroprotective effects, the optimal lower limit of UA levels may vary between the sexes. Therefore, sex differences in UA levels should be considered when predicting neurological outcomes after ischemic stroke.

The strengths of our study include the use of a large-scale multicenter registry, as well as the accurate diagnosis of stroke and its recurrence due to high rates of magnetic resonance imaging usage (98.5%). Moreover, the precise evaluation of changes in neurological severity may help to accurately determine the associations of UA levels with neurological improvement and deterioration. However, this study has several limitations. First, the number and timing of blood tests was not standardized among patients, which may have contributed to the misclassification of UA decrease rate grades. We performed a sensitivity analysis that focused on patients admitted within 24 hours of stroke onset to remove this uncertainty and validate our results in groups under similar conditions. Second, a large number of patients had a single measurement of UA during hospitalization and were excluded as cases with missing data (S9 Table). When compared with the study patients, the excluded patients generally had lower NIHSS scores on admission and a higher proportion of neurological improvement. Therefore, the exclusion of these patients could have introduced selection bias that skewed our study toward individuals with a more severe condition. Third, we did not evaluate body fluid volume, infusion volume, or infarct size in this study. As an alternative to infarct size, we included neurological severity (NIHSS on admission) as an adjustment factor in the multivariable analyses. Fourth, our results only describe an association between decreases in UA levels and poor functional outcomes, and do not establish a causal relationship. Thus, we are unable to determine the benefits and risks of administering anti-hyperuricemic agents or UA itself. Further studies are needed to investigate the impact of raising UA levels on outcomes. Finally, our findings are limited in generalizability because the participating hospitals were restricted to a single region of Japan.

## Conclusions

Decreases in serum UA levels were found to be independently associated with unfavorable short-term outcomes of acute ischemic stroke. Further analyses are required to verify the underlying molecular mechanisms and to determine interventions for maintaining serum UA levels in ischemic stroke patients.

## Supporting information

S1 Fig Flow chart of patient selection. An outlier uric acid level was defined as a concentration of >1784 μmol/L (30 mg/dL). mRS indicates modified Rankin Scale.
(PDF)

S2 Fig Subgroup analyses of the association between decreases in serum UA levels and functional dependence at 3 months. The ORs and 95% CIs of functional dependence (defined as an mRS score of 3–5 at 3 months) are shown according to serum UA decrease rate grade (G1 to G4) in each subgroup. The subgroups included (**A**) age (<75 or ≥75 years), (**B**) sex (women or men), (**C**) stroke subtype (non-cardioembolic or cardioembolic), (**D**) neurological severity (NIHSS <5 or ≥5), (**E**) CKD (presence or absence), and (**F**) UA level on admission (<292 or ≥292 μmol/L for women, <351 or ≥351 μmol/L for men). The multivariable models adjusted for patient age, sex, modified Rankin Scale score before stroke onset, body mass index, acute reperfusion therapy, NIHSS score on admission, stroke subtype, hypertension, diabetes mellitus, dyslipidemia, atrial fibrillation, smoking habit, alcohol habit, estimated glomerular filtration rate, length of hospital stay, and serum UA level on admission. *P* values for heterogeneity (*P*h) were calculated by adding the interaction term of UA decrease rate grade × subgroup to the multivariable models. CI indicates confidence interval; CKD, chronic kidney disease; NIHSS, National Institutes of Health Stroke Scale; OR, odds ratio; and UA, uric acid.
(PDF)

S3 Fig Subgroup analyses of the association between decreases in serum UA levels and neurological improvement during hospitalization. The ORs and 95% CIs of neurological improvement (defined as a ≥4-point decrease in NIHSS score during hospitalization or a score of zero at discharge) are shown according to serum UA decrease rate grade (G1 to G4) in each subgroup. The subgroups included (**A**) age (<75 or ≥75 years), (**B**) sex (women or men), (**C**) stroke subtype (non-cardioembolic or cardioembolic), (**D**) neurological severity (NIHSS <5 or ≥5), (**E**) CKD (presence or absence), and (**F**) UA level on admission (<292 or ≥292 μmol/L for women, <351 or ≥351 μmol/L for men). The multivariable models adjusted for patient age, sex, modified Rankin Scale score before stroke onset, body mass index, acute reperfusion therapy, NIHSS score on admission, stroke subtype, hypertension, diabetes mellitus, dyslipidemia, atrial fibrillation, smoking habit, alcohol habit, estimated glomerular filtration rate, length of hospital stay, and serum UA level on admission. *P* values for heterogeneity (*P*h) were calculated by adding the interaction term of UA decrease rate grade × subgroup to the multivariable models. CI indicates confidence interval; CKD, chronic kidney disease; NIHSS, National Institutes of Health Stroke Scale; OR, odds ratio; and UA, uric acid.
(PDF)

S4 Fig Subgroup analyses of the association between decreases in serum UA levels and neurological deterioration during hospitalization. The ORs and 95% CIs of neurological deterioration (defined as a ≥1-point increase in the NIHSS score during hospitalization) are shown according to serum UA decrease rate grade (G1 to G4) in each subgroup. The subgroups included (**A**) age (<75 or ≥75 years), (**B**) sex (women or men), (**C**) stroke subtype (non-cardioembolic or cardioembolic), (**D**) neurological severity (NIHSS <5 or ≥5), (**E**) CKD (presence or absence), and (**F**) UA level on admission (<292 or ≥292 μmol/L for women, <351 or ≥351 μmol/L for men). The multivariable models adjusted for patient age, sex, modified Rankin Scale score before stroke onset, body mass index, acute reperfusion therapy, NIHSS score on admission, stroke subtype, hypertension, diabetes mellitus, dyslipidemia, atrial fibrillation, smoking habit, alcohol habit, estimated glomerular filtration rate, length of hospital stay, and serum UA level on admission. *P* values for heterogeneity (*P*h) were calculated by adding the interaction term of UA decrease rate grade × subgroup to the multivariable models. CI indicates confidence interval; CKD, chronic kidney disease; NIHSS, National Institutes of Health Stroke Scale; OR, odds ratio; and UA, uric acid.
(PDF)

S1 Table Associations between decreases in serum UA levels and functional outcomes at 3 months. Poor functional outcome and functional dependence were defined as mRS scores of 2–6 and 2–5, respectively, at 3 months after stroke onset. G1 to G4 indicate the grades of serum UA decrease rates. The multivariable models adjusted for patient age, sex, modified Rankin Scale score before stroke onset, body mass index, acute reperfusion therapy, National Institutes of Health Stroke Scale score on admission, stroke subtype, hypertension, diabetes mellitus, dyslipidemia, atrial fibrillation, smoking habit, alcohol habit, estimated glomerular filtration rate, length of hospital stay, and serum UA level on admission. CI indicates confidence interval; OR, odds ratio; *P*_trend_, *P* for trend; and UA, uric acid.
(PDF)

S2 Table Associations between decreases in serum UA levels (from admission to nearest to discharge) and functional outcomes at 3 months. Poor functional outcome and functional dependence were defined as mRS scores of 3–6 and 3–5, respectively, at 3 months after stroke onset. Q1 to Q4 indicate the quartiles of serum UA decrease rates from admission to nearest to discharge. The multivariable models adjusted for patient age, sex, modified Rankin Scale score before stroke onset, body mass index, acute reperfusion therapy, National Institutes of Health Stroke Scale score on admission, stroke subtype, hypertension, diabetes mellitus, dyslipidemia, atrial fibrillation, smoking habit, alcohol habit, estimated glomerular filtration rate, length of hospital stay, and serum UA level on admission. CI indicates confidence interval; OR, odds ratio; *P*_trend_, *P* for trend; and UA, uric acid.
(PDF)

S3 Table Associations between decreases in serum UA levels and all-cause death/stroke recurrence within 3 months. G1 to G4 indicate the grades of serum UA decrease rates. The multivariable models adjusted for patient age, sex, modified Rankin Scale score before stroke onset, body mass index, acute reperfusion therapy, National Institutes of Health Stroke Scale score on admission, stroke subtype, hypertension, diabetes mellitus, dyslipidemia, atrial fibrillation, smoking habit, alcohol habit, estimated glomerular filtration rate, length of hospital stay, and serum UA level on admission. CI indicates confidence interval; OR, odds ratio; *P*_trend_, *P* for trend; and UA, uric acid.
(PDF)

S4 Table Patient characteristics according to serum UA levels on admission. The serum UA levels on admission were categorized into sex-specific quintiles. BMI indicates body mass index; eGFR, estimated glomerular filtration rate; IQR, interquartile range; NIHSS, National Institutes of Health Stroke Scale; *P*_trend_, *P* for trend; SD, standard deviation; and UA, uric acid.
(PDF)

S5 Table Associations between serum UA levels on admission and functional outcomes at 3 months. Poor functional outcome and functional dependence were defined as mRS scores of 3–6 and 3–5, respectively, at 3 months after stroke onset. Q1 to Q5 indicate the quintiles of serum UA levels on admission. The multivariable models adjusted for patient age, sex, modified Rankin Scale score before stroke onset, body mass index, acute reperfusion therapy, National Institutes of Health Stroke Scale score on admission, stroke subtype, hypertension, diabetes mellitus, dyslipidemia, atrial fibrillation, smoking habit, alcohol habit, estimated glomerular filtration rate, and length of hospital stay. CI indicates confidence interval; OR, odds ratio; *P*_trend_, *P* for trend; and UA, uric acid.
(PDF)

S6 Table Associations between decreases in serum UA levels and functional outcomes at 3 months among patients admitted within 24 hours of stroke onset. Poor functional outcome and functional dependence were defined as mRS scores of 3–6 and 3–5, respectively, at 3 months after stroke onset. G1 to G4 indicate the grades of serum UA decrease rates. The multivariable models adjusted for patient age, sex, modified Rankin Scale score before stroke onset, body mass index, acute reperfusion therapy, National Institutes of Health Stroke Scale score on admission, stroke subtype, hypertension, diabetes mellitus, dyslipidemia, atrial fibrillation, smoking habit, alcohol habit, estimated glomerular filtration rate, length of hospital stay, and serum UA level on admission. CI indicates confidence interval; OR, odds ratio; *P*_trend_, *P* for trend; and UA, uric acid.
(PDF)

S7 Table Associations between decreases in serum UA levels and functional outcomes at 3 months among patients who were not administered antihyperuricemics during hospitalization. Poor functional outcome and functional dependence were defined as mRS scores of 3–6 and 3–5, respectively, at 3 months after stroke onset. G1 to G4 indicate the grades of serum UA decrease rates. The multivariable models adjusted for patient age, sex, modified Rankin Scale score before stroke onset, body mass index, acute reperfusion therapy, National Institutes of Health Stroke Scale score on admission, stroke subtype, hypertension, diabetes mellitus, dyslipidemia, atrial fibrillation, smoking habit, alcohol habit, estimated glomerular filtration rate, length of hospital stay, and serum UA level on admission. CI indicates confidence interval; OR, odds ratio; *P*_trend_, *P* for trend; and UA, uric acid.
(PDF)

S8 Table Associations between decreases in serum UA levels and functional outcomes at 3 months after adjusting for serum albumin and hematocrit levels on admission. A total of 560 patients were excluded from these analyses due to missing data in serum albumin levels. Poor functional outcome and functional dependence were defined as mRS scores of 3–6 and 3–5, respectively, at 3 months after stroke onset. G1 to G4 indicate the grades of serum UA decrease rates. The multivariable models adjusted for patient age, sex, modified Rankin Scale score before stroke onset, body mass index, acute reperfusion therapy, National Institutes of Health Stroke Scale score on admission, stroke subtype, hypertension, diabetes mellitus, dyslipidemia, atrial fibrillation, smoking habit, alcohol habit, estimated glomerular filtration rate, length of hospital stay, serum UA level on admission, serum albumin level on admission, and hematocrit level on admission. CI indicates confidence interval; OR, odds ratio; *P*_trend_, *P* for trend; and UA, uric acid.
(PDF)

S9 Table Patient characteristics of cases excluded due to missing data in serum UA levels on admission. Poor functional outcome and functional dependence were defined as mRS scores of 3–6 and 3–5, respectively, at 3 months after stroke onset. Neurological improvement and neurological deterioration were defined as a ≥4-point decrease in NIHSS score during hospitalization or a score of zero at discharge and a ≥1-point increase in NIHSS score during hospitalization, respectively. *The numbers of study patients and patients with missing UA data were 4,061 and 5,700, respectively, after excluding those with missing data in serum albumin levels. BMI indicates body mass index; eGFR, estimated glomerular filtration rate; IQR, interquartile range; NIHSS, National Institutes of Health Stroke Scale; *P*_trend_, *P* for trend; SD, standard deviation; and UA, uric acid.
(PDF)
