# Supplementary material for: Association between decreases in serum uric acid levels and unfavorable outcomes after ischemic stroke: A multicenter hospital-based observational study
Source: PLoS One. 2023 Jun 29;18(6):e0287721. doi: 10.1371/journal.pone.0287721 (PMC10309981; doi:10.1371/journal.pone.0287721)
Supplement: S1 Fig — An outlier uric acid level was defined as a concentration of >1784 μmol/L (30 mg/dL). mRS indicates modified Rankin Scale. (PDF) [file pone.0287721.s001.pdf]

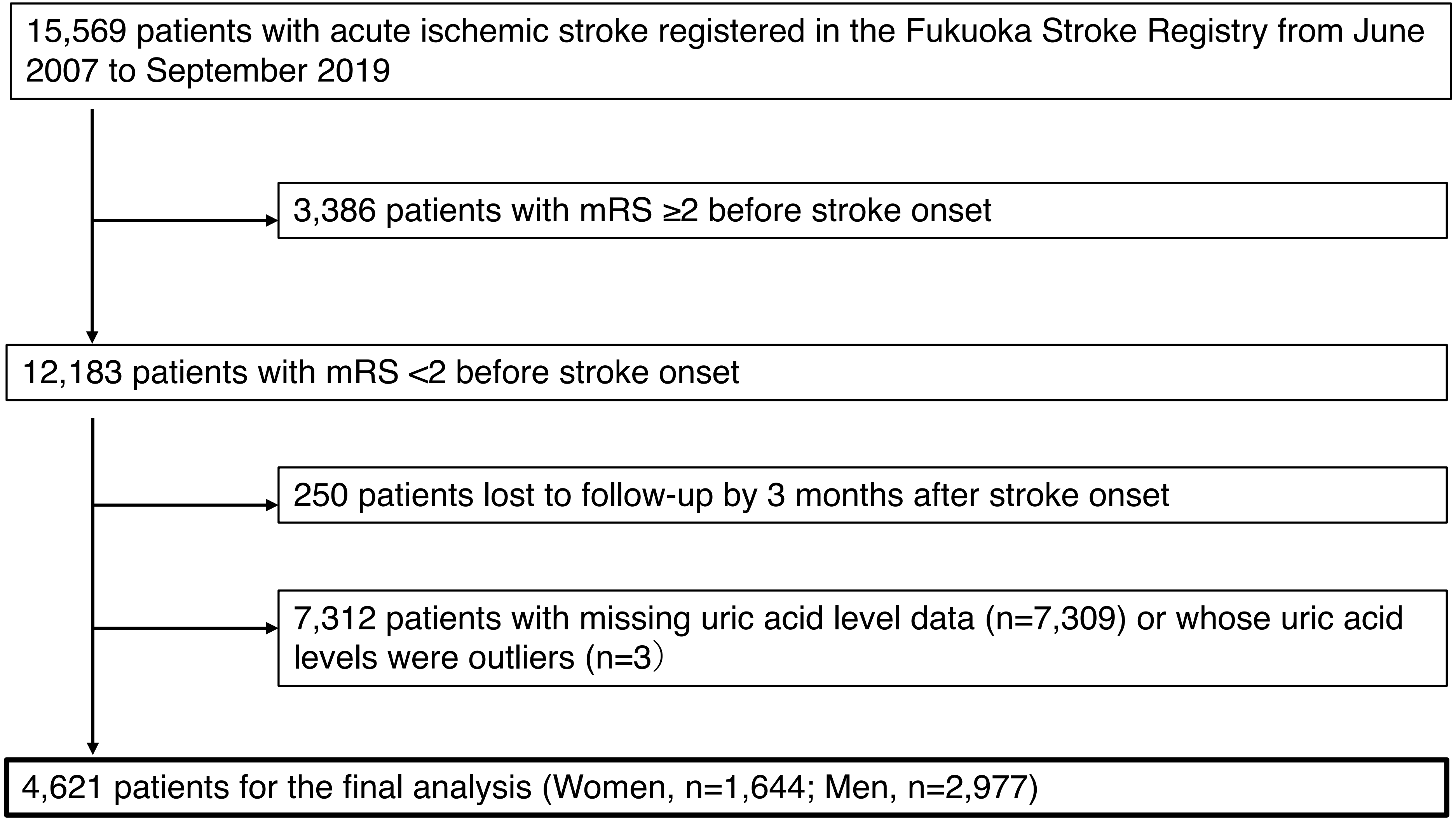
15,569 patients with acute ischemic stroke registered in the Fukuoka Stroke Registry from June 2007 to September 2019
3,386 patients with mRS ≥2 before stroke onset
12,183 patients with mRS <2 before stroke onset
250 patients lost to follow-up by 3 months after stroke onset
7,312 patients with missing uric acid level data (n=7,309) or whose uric acid levels were outliers (n=3）
4,621 patients for the final analysis (Women, n=1,644; Men, n=2,977)
