# Supplementary figures and images for: Association between decreases in serum uric acid levels and unfavorable outcomes after ischemic stroke: A multicenter hospital-based observational study
Source: PLoS One. 2023 Jun 29;18(6):e0287721. doi: 10.1371/journal.pone.0287721 (PMC10309981; doi:10.1371/journal.pone.0287721)

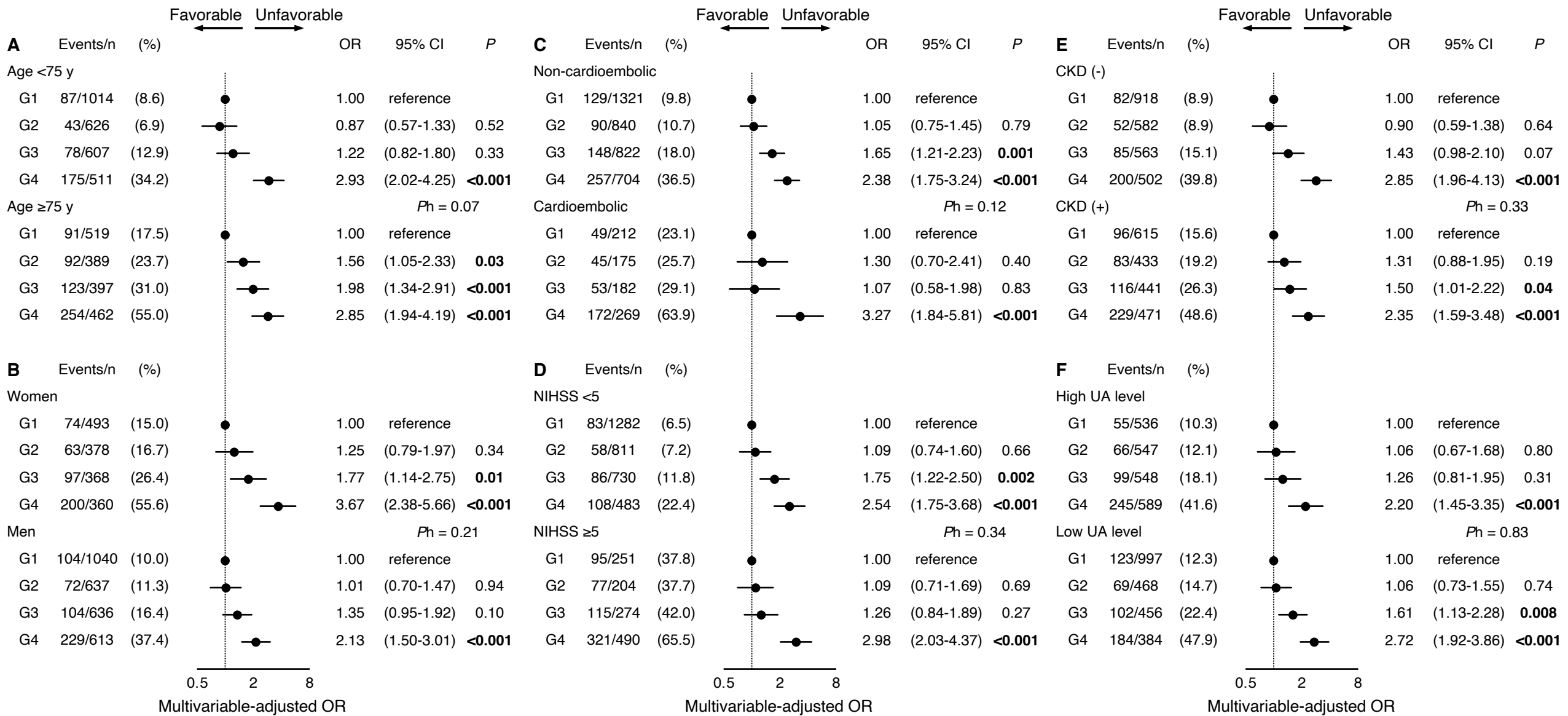

Supplement: S2 Fig — The ORs and 95% CIs of functional dependence (defined as an mRS score of 3–5 at 3 months) are shown according to serum UA decrease rate grade (G1 to G4) in each subgroup. The subgroups included (A) age (<75 or ≥75 years), (B) sex (women or men), (C) stroke subtype (non-cardioembolic or cardioembolic), (D) neurological severity (NIHSS <5 or ≥5), (E) CKD (presence or absence), and (F) UA level on admission (<292 or ≥292 μmol/L for women, <351 or ≥351 μmol/L for men). The multivariable models adjusted for patient age, sex, modified Rankin Scale score before stroke onset, body mass index, acute reperfusion therapy, NIHSS score on admission, stroke subtype, hypertension, diabetes mellitus, dyslipidemia, atrial fibrillation, smoking habit, alcohol habit, estimated glomerular filtration rate, length of hospital stay, and serum UA level on admission. P values for heterogeneity (Ph) were calculated by adding the interaction term of UA decrease rate grade × subgroup to the multivariable models. CI indicates confidence interval; CKD, chronic kidney disease; NIHSS, National Institutes of Health Stroke Scale; OR, odds ratio; and UA, uric acid. (PDF) [file pone.0287721.s002.pdf]

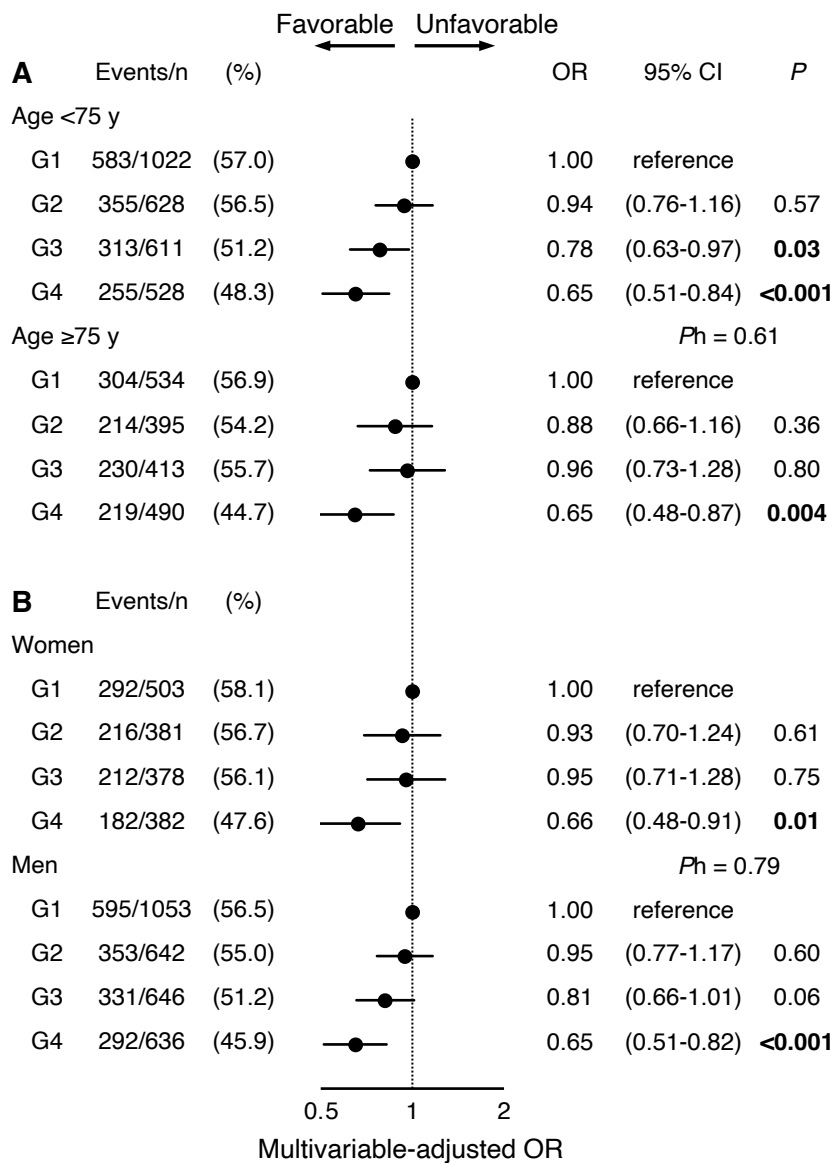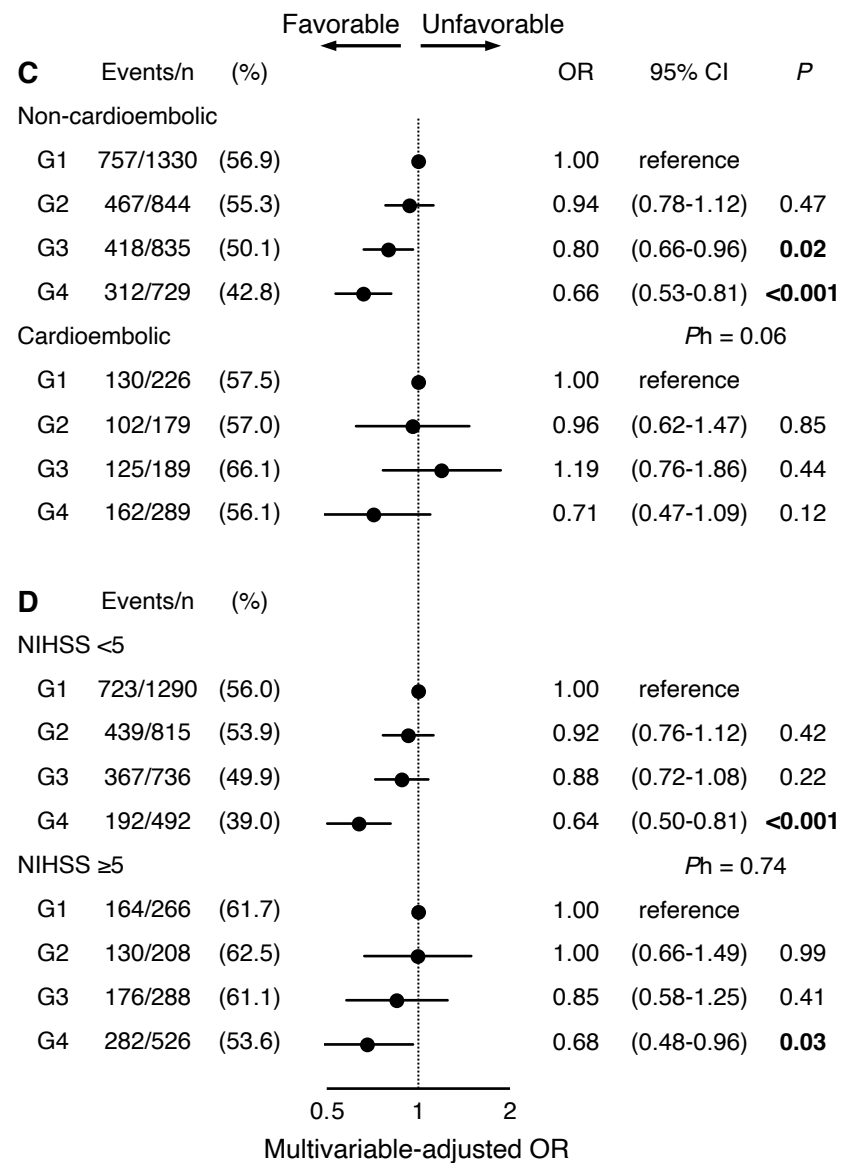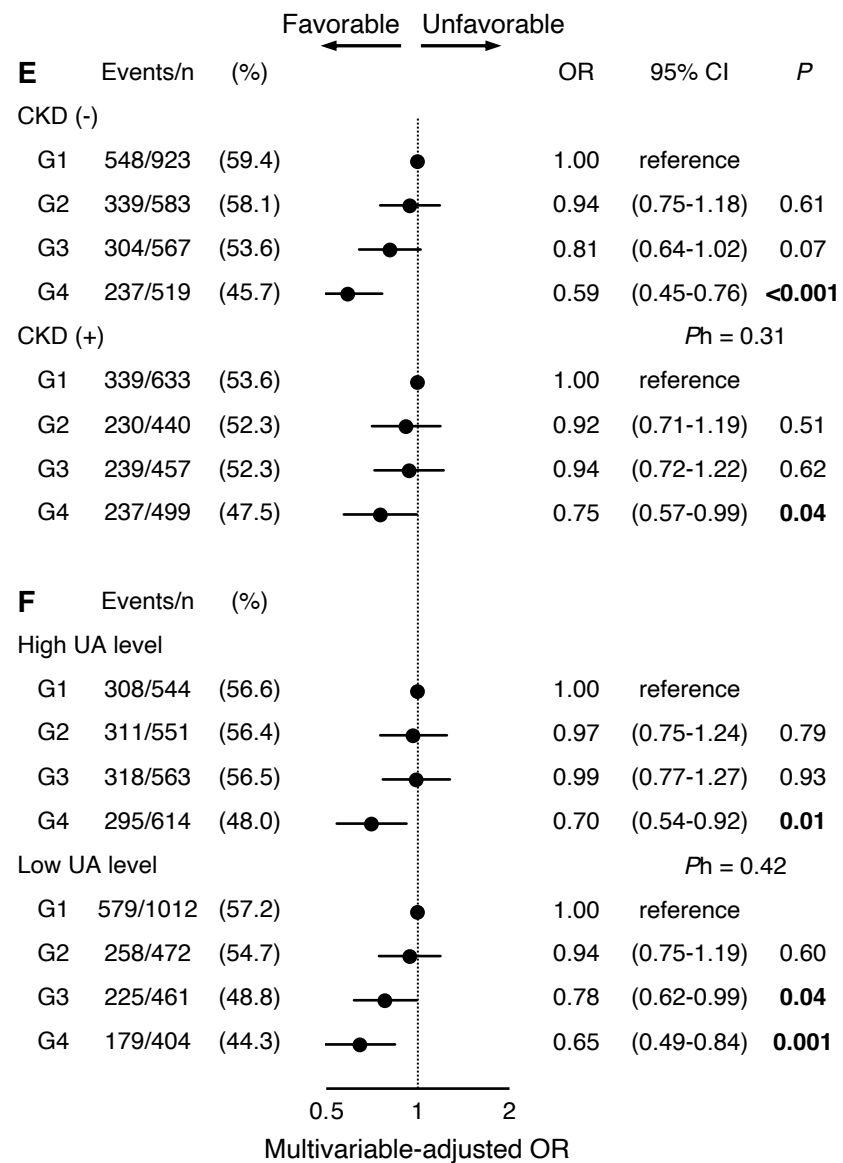

Supplement: S3 Fig — The ORs and 95% CIs of neurological improvement (defined as a ≥4-point decrease in NIHSS score during hospitalization or a score of zero at discharge) are shown according to serum UA decrease rate grade (G1 to G4) in each subgroup. The subgroups included (A) age (<75 or ≥75 years), (B) sex (women or men), (C) stroke subtype (non-cardioembolic or cardioembolic), (D) neurological severity (NIHSS <5 or ≥5), (E) CKD (presence or absence), and (F) UA level on admission (<292 or ≥292 μmol/L for women, <351 or ≥351 μmol/L for men). The multivariable models adjusted for patient age, sex, modified Rankin Scale score before stroke onset, body mass index, acute reperfusion therapy, NIHSS score on admission, stroke subtype, hypertension, diabetes mellitus, dyslipidemia, atrial fibrillation, smoking habit, alcohol habit, estimated glomerular filtration rate, length of hospital stay, and serum UA level on admission. P values for heterogeneity (Ph) were calculated by adding the interaction term of UA decrease rate grade × subgroup to the multivariable models. CI indicates confidence interval; CKD, chronic kidney disease; NIHSS, National Institutes of Health Stroke Scale; OR, odds ratio; and UA, uric acid. (PDF) [file pone.0287721.s003.pdf]

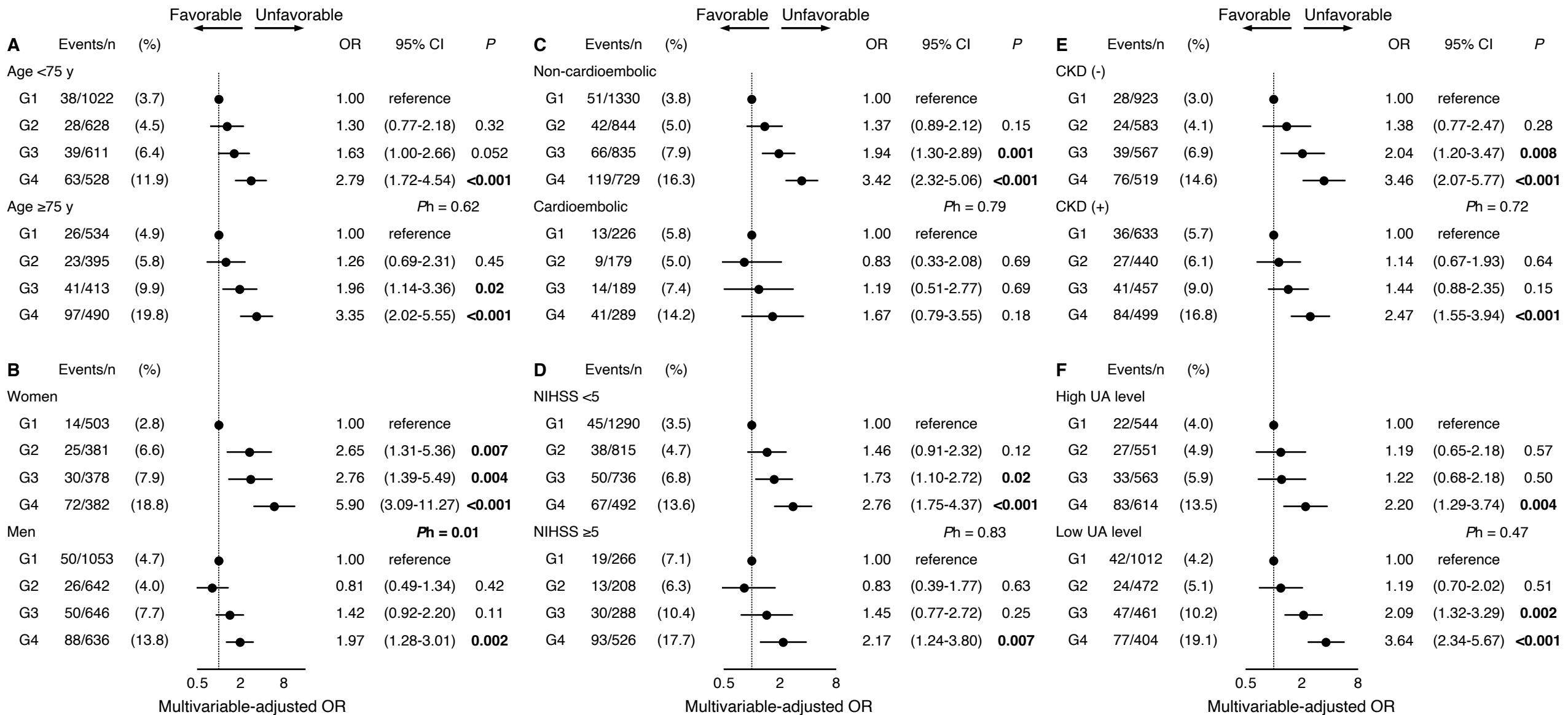

Supplement: S4 Fig — The ORs and 95% CIs of neurological deterioration (defined as a ≥1-point increase in the NIHSS score during hospitalization) are shown according to serum UA decrease rate grade (G1 to G4) in each subgroup. The subgroups included (A) age (<75 or ≥75 years), (B) sex (women or men), (C) stroke subtype (non-cardioembolic or cardioembolic), (D) neurological severity (NIHSS <5 or ≥5), (E) CKD (presence or absence), and (F) UA level on admission (<292 or ≥292 μmol/L for women, <351 or ≥351 μmol/L for men). The multivariable models adjusted for patient age, sex, modified Rankin Scale score before stroke onset, body mass index, acute reperfusion therapy, NIHSS score on admission, stroke subtype, hypertension, diabetes mellitus, dyslipidemia, atrial fibrillation, smoking habit, alcohol habit, estimated glomerular filtration rate, length of hospital stay, and serum UA level on admission. P values for heterogeneity (Ph) were calculated by adding the interaction term of UA decrease rate grade × subgroup to the multivariable models. CI indicates confidence interval; CKD, chronic kidney disease; NIHSS, National Institutes of Health Stroke Scale; OR, odds ratio; and UA, uric acid. (PDF) [file pone.0287721.s004.pdf]
