# Supplementary material for: Association between decreases in serum uric acid levels and unfavorable outcomes after ischemic stroke: A multicenter hospital-based observational study
Source: PLoS One. 2023 Jun 29;18(6):e0287721. doi: 10.1371/journal.pone.0287721 (PMC10309981; doi:10.1371/journal.pone.0287721)
Supplement: S4 Table — The serum UA levels on admission were categorized into sex-specific quintiles. BMI indicates body mass index; eGFR, estimated glomerular filtration rate; IQR, interquartile range; NIHSS, National Institutes of Health Stroke Scale; Ptrend, P for trend; SD, standard deviation; and UA, uric acid. (PDF) [file pone.0287721.s008.pdf]

**S4 Table. Patient characteristics according to serum UA levels on admission.**

| | Q1 | Q2 | Q3 | Q4 | Q5 | $P_{trend}$ |
|---|---|---|---|---|---|---|
| UA level (μmol/L), women | ≤228 | 229–270 | 271–312 | 313–365 | >365 | |
| UA level (μmol/L), men | ≤276 | 277–324 | 325–371 | 372–431 | >431 | |
| n | 913 | 864 | 934 | 954 | 956 | |
| Age (years), mean ± SD | 71.1±11.6 | 69.6±12.0 | 70.0±12.4 | 70.4±11.4 | 69.2±13.5 | 0.08 |
| Men, n (%) | 572 (62.7) | 558 (64.6) | 612 (65.5) | 623 (65.3) | 612 (64.0) | 0.49 |
| BMI (kg/m$^2$), mean ± SD | 22.3±3.4 | 23.1±3.3 | 23.4±3.6 | 23.7±3.5 | 24.2±4.4 | **<0.001** |
| eGFR (mL/min/1.73 m$^2$), mean ± SD | 74.5±28.7 | 71.2±24.3 | 68.1±21.6 | 62.1±21.7 | 54.7±22.8 | **<0.001** |
| Risk factors, n (%) | | | | | | |
| Hypertension | 690 (75.6) | 691 (80.0) | 752 (80.5) | 820 (86.0) | 844 (88.3) | **<0.001** |
| Diabetes mellitus | 355 (38.9) | 334 (38.7) | 272 (29.1) | 278 (29.1) | 287 (30.0) | **<0.001** |
| Dyslipidemia | 505 (55.3) | 499 (57.8) | 545 (58.4) | 594 (62.3) | 587 (61.4) | **0.001** |
| Atrial fibrillation | 189 (20.7) | 157 (18.2) | 174 (18.6) | 221 (23.2) | 247 (25.8) | **<0.001** |
| Smoking habit | 528 (57.8) | 497 (57.5) | 531 (56.9) | 571 (59.9) | 589 (61.6) | 0.051 |
| Alcohol habit | 307 (33.6) | 300 (34.7) | 365 (39.1) | 397 (41.6) | 400 (41.8) | **<0.001** |
| Coronary artery disease, n (%) | 137 (15.0) | 122 (14.1) | 118 (12.6) | 137 (14.4) | 153 (16.0) | 0.52 |
| Chronic kidney disease, n (%) | 272 (29.8) | 305 (35.3) | 367 (39.3) | 466 (48.8) | 619 (64.7) | **<0.001** |
| Previous history of stroke, n (%) | 130 (14.2) | 137 (15.9) | 143 (15.3) | 173 (18.1) | 143 (15.0) | 0.33 |
| Stroke subtypes, n (%) | | | | | | |
| Cardioembolism | 179 (19.6) | 132 (15.3) | 162 (17.3) | 186 (19.5) | 224 (23.4) | **0.004** |
| Large artery atherosclerosis | 137 (15.0) | 140 (16.2) | 186 (19.9) | 170 (17.8) | 155 (16.2) | |
| Small vessel occlusion | 282 (30.9) | 296 (34.3) | 279 (29.9) | 278 (29.1) | 256 (26.8) | |
| Other causes | 315 (34.5) | 296 (34.3) | 307 (32.9) | 320 (33.5) | 321 (33.6) | |
| Reperfusion therapy, n (%) | 105 (11.5) | 97 (11.2) | 122 (13.1) | 125 (13.1) | 125 (13.1) | 0.15 |
| NIHSS score on admission, median (IQR) | 3 (1-5) | 2 (1-4) | 2 (1-5) | 2 (1-5) | 3 (1-6) | 0.71 |
| Length of hospital stay (days), median (IQR) | 18 (13-26) | 17 (12.25-25) | 18 (12-25) | 18 (12-25) | 19 (13-27) | 0.71 |
| Medication, n (%) | | | | | | |
| Antihyperuricemic use during hospitalization | 55 (6.0) | 68 (7.9) | 86 (9.2) | 114 (12.0) | 199 (20.8) | **<0.001** |

The serum UA levels on admission were categorized into sex-specific quintiles.

BMI indicates body mass index; eGFR, estimated glomerular filtration rate; IQR, interquartile range; NIHSS, National Institutes of Health Stroke Scale; $P_{trend}$, $P$ for trend; SD, standard deviation; and UA, uric acid.
