# Supplementary material for: Association between decreases in serum uric acid levels and unfavorable outcomes after ischemic stroke: A multicenter hospital-based observational study
Source: PLoS One. 2023 Jun 29;18(6):e0287721. doi: 10.1371/journal.pone.0287721 (PMC10309981; doi:10.1371/journal.pone.0287721)
Supplement: S5 Table — Poor functional outcome and functional dependence were defined as mRS scores of 3–6 and 3–5, respectively, at 3 months after stroke onset. Q1 to Q5 indicate the quintiles of serum UA levels on admission. The multivariable models adjusted for patient age, sex, modified Rankin Scale score before stroke onset, body mass index, acute reperfusion therapy, National Institutes of Health Stroke Scale score on admission, stroke subtype, hypertension, diabetes mellitus, dyslipidemia, atrial fibrillation, smoking habit, alcohol habit, estimated glomerular filtration rate, and length of hospital stay. CI indicates confidence interval; OR, odds ratio; Ptrend, P for trend; and UA, uric acid. (PDF) [file pone.0287721.s009.pdf]

S5 Table. Associations between serum UA levels on admission and functional outcomes at 3 months.

	Events/total (%)		Age- and sex-adjusted				Multivariable-adjusted			
			OR	95% CI	*P*	P_{trend}	OR	95% CI	*P*	P_{trend}
**Poor functional outcome at 3 months (mRS score of 3–6)**										
Q1	218/913	(23.9)	1.00	reference		0.59	1.00	reference		0.45
Q2	179/864	(20.7)	0.90	(0.71-1.14)	0.37		1.09	(0.78-1.52)	0.61	
Q3	212/934	(22.7)	0.99	(0.79-1.24)	0.92		1.29	(0.87-1.91)	0.21	
Q4	198/954	(20.8)	0.87	(0.69-1.09)	0.21		1.17	(0.72-1.91)	0.52	
Q5	232/956	(24.3)	1.09	(0.87-1.37)	0.43		1.28	(0.64-2.55)	0.49	
**Functional dependence at 3 months (mRS score of 3–5)**										
Q1	195/890	(21.9)	1.00	reference		0.68	1.00	reference		0.50
Q2	168/853	(19.7)	0.94	(0.74-1.19)	0.61		1.13	(0.80-1.59)	0.49	
Q3	194/916	(21.2)	1.01	(0.80-1.28)	0.92		1.31	(0.88-1.96)	0.19	
Q4	179/935	(19.1)	0.88	(0.70-1.11)	0.29		1.18	(0.71-1.95)	0.52	
Q5	207/931	(22.2)	1.09	(0.87-1.38)	0.46		1.24	(0.61-2.53)	0.55	

Poor functional outcome and functional dependence were defined as mRS scores of 3–6 and 3–5, respectively, at 3 months after stroke onset. Q1 to Q5 indicate the quintiles of serum UA levels on admission. The multivariable models adjusted for patient age, sex, modified Rankin Scale score before stroke onset, body mass index, acute reperfusion therapy, National Institutes of Health Stroke Scale score on admission, stroke subtype, hypertension, diabetes mellitus, dyslipidemia, atrial fibrillation, smoking habit, alcohol habit, estimated glomerular filtration rate, and length of hospital stay.

CI indicates confidence interval; OR, odds ratio; P_{trend}, *P* for trend; and UA, uric acid.
