# Supplementary material for: Association between decreases in serum uric acid levels and unfavorable outcomes after ischemic stroke: A multicenter hospital-based observational study
Source: PLoS One. 2023 Jun 29;18(6):e0287721. doi: 10.1371/journal.pone.0287721 (PMC10309981; doi:10.1371/journal.pone.0287721)
Supplement: S7 Table — Poor functional outcome and functional dependence were defined as mRS scores of 3–6 and 3–5, respectively, at 3 months after stroke onset. G1 to G4 indicate the grades of serum UA decrease rates. The multivariable models adjusted for patient age, sex, modified Rankin Scale score before stroke onset, body mass index, acute reperfusion therapy, National Institutes of Health Stroke Scale score on admission, stroke subtype, hypertension, diabetes mellitus, dyslipidemia, atrial fibrillation, smoking habit, alcohol habit, estimated glomerular filtration rate, length of hospital stay, and serum UA level on admission. CI indicates confidence interval; OR, odds ratio; Ptrend, P for trend; and UA, uric acid. (PDF) [file pone.0287721.s011.pdf]

**S7 Table. Associations between decreases in serum UA levels and functional outcomes at 3 months among patients who were not administered antihyperuricemics during hospitalization.**

| | Events/total (%) | | Age- and sex-adjusted | | | | Multivariable-adjusted | | | |
|---|---|---|---|---|---|---|---|---|---|---|
| | | | **OR** | **95% CI** | ***P*** | **$P_{trend}$** | **OR** | **95% CI** | ***P*** | **$P_{trend}$** |
| **Poor functional outcome at 3 months** | | | | | | | | | | |
| G1 | 182/1375 | (13.2) | 1.00 | reference | | **<0.001** | 1.00 | reference | | **<0.001** |
| G2 | 132/930 | (14.2) | 0.97 | (0.76-1.25) | 0.82 | | 1.07 | (0.80-1.43) | 0.66 | |
| G3 | 200/919 | (21.8) | 1.72 | (1.37-2.16) | **<0.001** | | 1.50 | (1.13-1.97) | **0.005** | |
| G4 | 435/875 | (49.7) | 5.76 | (4.65-7.13) | **<0.001** | | 2.76 | (2.10-3.63) | **<0.001** | |
| **Functional dependence at 3 months** | | | | | | | | | | |
| G1 | 160/1353 | (11.8) | 1.00 | reference | | **<0.001** | 1.00 | reference | | **<0.001** |
| G2 | 124/922 | (13.4) | 1.04 | (0.80-1.35) | 0.76 | | 1.11 | (0.82-1.51) | 0.49 | |
| G3 | 180/899 | (20.0) | 1.77 | (1.39-2.25) | **<0.001** | | 1.46 | (1.10-1.95) | **0.01** | |
| G4 | 391/831 | (47.1) | 5.85 | (4.69-7.31) | **<0.001** | | 2.71 | (2.04-3.60) | **<0.001** | |

Poor functional outcome and functional dependence were defined as mRS scores of 3–6 and 3–5, respectively, at 3 months after stroke onset. G1 to G4 indicate the grades of serum UA decrease rates. The multivariable models adjusted for patient age, sex, modified Rankin Scale score before stroke onset, body mass index, acute reperfusion therapy, National Institutes of Health Stroke Scale score on admission, stroke subtype, hypertension, diabetes mellitus, dyslipidemia, atrial fibrillation, smoking habit, alcohol habit, estimated glomerular filtration rate, length of hospital stay, and serum UA level on admission.

CI indicates confidence interval; OR, odds ratio; $P_{trend}$, *P* for trend; and UA, uric acid.
