# Supplementary material for: Association between decreases in serum uric acid levels and unfavorable outcomes after ischemic stroke: A multicenter hospital-based observational study
Source: PLoS One. 2023 Jun 29;18(6):e0287721. doi: 10.1371/journal.pone.0287721 (PMC10309981; doi:10.1371/journal.pone.0287721)
Supplement: S8 Table — A total of 560 patients were excluded from these analyses due to missing data in serum albumin levels. Poor functional outcome and functional dependence were defined as mRS scores of 3–6 and 3–5, respectively, at 3 months after stroke onset. G1 to G4 indicate the grades of serum UA decrease rates. The multivariable models adjusted for patient age, sex, modified Rankin Scale score before stroke onset, body mass index, acute reperfusion therapy, National Institutes of Health Stroke Scale score on admission, stroke subtype, hypertension, diabetes mellitus, dyslipidemia, atrial fibrillation, smoking habit, alcohol habit, estimated glomerular filtration rate, length of hospital stay, serum UA level on admission, serum albumin level on admission, and hematocrit level on admission. CI indicates confidence interval; OR, odds ratio; Ptrend, P for trend; and UA, uric acid. (PDF) [file pone.0287721.s012.pdf]

S8 Table. Associations between decreases in serum UA levels and functional outcomes at 3 months after adjusting for serum albumin and hematocrit levels on admission.

	Events/total (%)		Age- and sex-adjusted				Multivariable-adjusted			
			**OR**	**95% CI**	***P***	***P*trend**	**OR**	**95% CI**	***P***	***P*trend**
**Poor functional outcome at 3 months**										
G1	172/1369	(12.6)	1.00	reference		**<0.001**	1.00	reference		**<0.001**
G2	131/901	(14.5)	1.08	(0.84-1.39)	0.56		1.14	(0.85-1.54)	0.39	
G3	188/897	(21.0)	1.74	(1.38-2.20)	**<0.001**		1.58	(1.19-2.09)	**0.002**	
G4	414/894	(46.3)	5.48	(4.42-6.81)	**<0.001**		2.74	(2.08-3.63)	**<0.001**	
**Functional dependence at 3 months**										
G1	152/1349	(11.3)	1.00	reference		**<0.001**	1.00	reference		**<0.001**
G2	124/894	(13.9)	1.16	(0.89-1.5)	0.27		1.20	(0.88-1.63)	0.25	
G3	172/881	(19.5)	1.81	(1.42-2.31)	**<0.001**		1.55	(1.16-2.09)	**0.003**	
G4	375/855	(43.9)	5.58	(4.46-6.99)	**<0.001**		2.69	(2.02-3.59)	**<0.001**	

A total of 560 patients were excluded from these analyses due to missing data in serum albumin levels.

Poor functional outcome and functional dependence were defined as mRS scores of 3–6 and 3–5, respectively, at 3 months after stroke onset. G1 to G4 indicate the grades of serum UA decrease rates. The multivariable models adjusted for patient age, sex, modified Rankin Scale score before stroke onset, body mass index, acute reperfusion therapy, National Institutes of Health Stroke Scale score on admission, stroke subtype, hypertension, diabetes mellitus, dyslipidemia, atrial fibrillation, smoking habit, alcohol habit, estimated glomerular filtration rate, length of hospital stay, serum UA level on admission, serum albumin level on admission, and hematocrit level on admission.

CI indicates confidence interval; OR, odds ratio; P_{trend}, *P* for trend; and UA, uric acid.
