# Supplementary material for: Association between decreases in serum uric acid levels and unfavorable outcomes after ischemic stroke: A multicenter hospital-based observational study
Source: PLoS One. 2023 Jun 29;18(6):e0287721. doi: 10.1371/journal.pone.0287721 (PMC10309981; doi:10.1371/journal.pone.0287721)
Supplement: S9 Table — Poor functional outcome and functional dependence were defined as mRS scores of 3–6 and 3–5, respectively, at 3 months after stroke onset. Neurological improvement and neurological deterioration were defined as a ≥4-point decrease in NIHSS score during hospitalization or a score of zero at discharge and a ≥1-point increase in NIHSS score during hospitalization, respectively. *The numbers of study patients and patients with missing UA data were 4,061 and 5,700, respectively, after excluding those with missing data in serum albumin levels. BMI indicates body mass index; eGFR, estimated glomerular filtration rate; IQR, interquartile range; NIHSS, National Institutes of Health Stroke Scale; Ptrend, P for trend; SD, standard deviation; and UA, uric acid. (PDF) [file pone.0287721.s013.pdf]

S9 Table. Patient characteristics of cases excluded due to missing data in serum UA levels on admission.

	Study patients	Patients with missing UA data	*P*
n	4621	7312	
Age (years), mean ± SD	70.1±12.2	70.5±12.1	0.06
Men, n (%)	2977 (64.4)	4651 (63.6)	0.37
BMI (kg/m^2), mean ± SD	23.4±3.7	23.3±3.7	0.17
eGFR (mL/min/1.73 m^2), mean ± SD	65.9±24.9	69.5±23.5	**<0.001**
Risk factors, n (%)			
Hypertension	3797 (82.2)	5757 (78.7)	**<0.001**
Diabetes mellitus	1526 (33.0)	2123 (29.0)	**<0.001**
Dyslipidemia	2730 (59.1)	4119 (56.3)	**0.003**
Atrial fibrillation	988 (21.4)	1613 (22.1)	0.38
Smoking habit	2716 (58.8)	4140 (56.6)	**0.02**
Alcohol habit	1769 (38.3)	2880 (39.4)	0.23
Coronary artery disease, n (%)	667 (14.4)	967 (13.2)	0.06
Chronic kidney disease, n (%)	2029 (43.9)	2657 (36.4)	**<0.001**
Previous history of stroke, n (%)	726 (15.7)	1110 (15.2)	0.43
Stroke subtypes, n (%)			
Cardioembolism	883 (19.1)	1401 (19.2)	0.94
Large artery atherosclerosis	788 (17.1)	1123 (15.4)	
Small vessel occlusion	1391 (30.1)	2147 (29.4)	
Other causes	1559 (33.7)	2641 (36.1)	
Reperfusion therapy, n (%)	574 (12.4)	698 (9.5)	**<0.001**
NIHSS score on admission, median (IQR)	2 (1-5)	2 (1-5)	**0.002**
Length of hospital stay (days), median (IQR)	18 (13-26)	17 (12-26)	**0.01**
Albumin level on admission (g/L)*, median (IQR)	41 (38-44)	41 (39-44)	**0.001**
Hematocrit level on admission (%), median (IQR)	40.8 (37.3-43.9)	41.0 (37.8-44.0)	**0.02**
Medication, n (%)			
Antihyperuricemic use during hospitalization	522 (11.3)	502 (6.9)	**<0.001**
Outcomes, n (%)			
Poor functional outcome at 3 months	1039 (22.5)	1612 (22.1)	0.59
Functional dependence at 3 months	943 (20.8)	1450 (20.3)	0.48
Neurological improvement during hospitalization	2473 (53.5)	4094 (56.0)	**0.008**
Neurological deterioration during hospitalization	355 (7.7)	556 (7.6)	0.88
Death in hospital	45 (1.0)	84 (1.1)	0.37

Poor functional outcome and functional dependence were defined as mRS scores of 3–6 and 3–5, respectively, at 3 months after stroke onset. Neurological improvement and neurological deterioration were defined as a ≥4-point decrease in NIHSS score during hospitalization or a score of zero at discharge and a ≥1-point increase in NIHSS score during hospitalization, respectively.

*The numbers of study patients and patients with missing UA data were 4,061 and 5,700, respectively, after excluding those with missing data in serum albumin levels.

BMI indicates body mass index; eGFR, estimated glomerular filtration rate; IQR, interquartile range; NIHSS, National Institutes of Health Stroke Scale; SD, standard deviation; and UA, uric acid.
